# Socioeconomic patterns in the use of public and private health services and equity in health care

**DOI:** 10.1186/1472-6963-8-183

**Published:** 2008-09-14

**Authors:** Enrique Regidor, David Martínez, María E Calle, Paloma Astasio, Paloma Ortega, Vicente Domínguez

**Affiliations:** 1 Department of Preventive Medicine and Public Health, Universidad Complutense de Madrid, Spain

## Abstract

**Background:**

Several studies in wealthy countries suggest that utilization of GP and hospital services, after adjusting for health care need, is equitable or pro-poor, whereas specialist care tends to favour the better off. Horizontal equity in these studies has not been evaluated appropriately, since the use of healthcare services is analysed without distinguishing between public and private services. The purpose of this study is to estimate the relation between socioeconomic position and health services use to determine whether the findings are compatible with the attainment of horizontal equity: equal use of public healthcare services for equal need.

**Methods:**

Data from a sample of 18,837 Spanish subjects were analysed to calculate the percentage of use of public and private general practitioner (GP), specialist and hospital care according to three indicators of socioeconomic position: educational level, social class and income. The percentage ratio was used to estimate the magnitude of the relation between each measure of socioeconomic position and the use of each health service.

**Results:**

After adjusting for age, sex and number of chronic diseases, a gradient was observed in the magnitude of the percentage ratio for public GP visits and hospitalisation: persons in the lowest socioeconomic position were 61–88% more likely to visit public GPs and 39–57% more likely to use public hospitalisation than those in the highest socioeconomic position. In general, the percentage ratio did not show significant socioeconomic differences in the use of public sector specialists. The magnitude of the percentage ratio in the use of the three private services also showed a socioeconomic gradient, but in exactly the opposite direction of the gradient observed in the public services.

**Conclusion:**

These findings show inequity in GP visits and hospitalisations, favouring the lower socioeconomic groups, and equity in the use of the specialist physician. These inequities could represent an overuse of public healthcare services or could be due to the fact that persons in high socioeconomic positions choose to use private health services.

## Background

Research on health services use in wealthy countries with public healthcare systems has yielded similar results. Studies in representative samples of the general population have found that, after controlling for health status, the probability of consulting a general practitioner (GP) and of hospitalisation either do not vary across income or socioeconomic groups, or is somewhat higher in persons belonging to the lowest socioeconomic groups [1-7]. In contrast, the probability of consulting the specialist physician is greater among higher-income and better-educated groups [1-3,7-9].

Based on these findings, it has been pointed out that these countries are generally characterized by the existence of equity or pro-poor inequity in GP visits and in hospitalisation, and of pro-rich inequity in visits to specialist physicians [7,10]. However, such judgements cannot be made based on the findings of these studies, since they analyse the use of healthcare services without distinguishing between the use of public and private services. Equity in the use of health services has an ethical dimension based on the social agreement according to which the objective of public healthcare systems, characterised by the extension of coverage to the entire population, is to achieve equal use of health care services for equal need. This is what is known as "horizontal equity" [11]. That is, if we assume an egalitarian distribution and adequate quality of public health resources, equity would be achieved when there is equality in the use of the public health services by all socioeconomic groups with an equal level of need [12].

Some of the reasons that have been offered to explain the pro-rich inequity in access to specialist services are the existence of complementary private health insurance companies and direct payments for health services [7,10,13]. It is true that private insurance policies make it possible for a certain proportion of the population to use private health services for which there is an alternative in the public health system. Given that persons of high socioeconomic position are more likely to have these policies, it has been observed that this situation may reduce equity [7,10,13]. However, there is no evidence that the use of private medical services alters the attainment of equity in the use of public health services [14]. A recent investigation of arthritis care in the United Kingdom showed that the National Health System achieved its equity goal of equal care for equal medical need, even though patients with more education were more likely to have private care [15].

The objective of this work was to estimate the relation between socioeconomic position and health services use in Spain, in order to evaluate whether the findings obtained are compatible with the achievement of horizontal equity. Specifically, we studied socioeconomic patterns in the use of three health services offered by the public health system and in the use of these same three services offered by the private sector.

## Methods

The data were taken from the 2003 Spanish National Health Survey carried out between April 2003 and March 2004. The data are publicly available accessing to web of Ministry of Health . Respondents were selected by stratified multistage sampling of the non-institutionalised population residing in Spain. The first stage units – census sections – were grouped into strata by size of the municipal population and were then selected with a probability proportional to the size of the population of the stratum. The second stage units – households – were selected within each census section with the same probability by systematic sampling with random start. Within each household one person aged 16 years or older was selected to complete the questionnaire. For the present analysis, we excluded persons older than 74 years because the probability of their being institutionalised increases after that age.

The three health services investigated were GP visits, specialist visits and hospitalisation. About 99.5% of the population residing in Spain has some type of public health coverage [16] and therefore has the right to use any of these services of the public health system. Citizens are registered with a public GP who is responsible for delivering primary care and who is the gatekeeper to public secondary care – specialists or hospitalisation. In general, patients cannot gain access to public secondary care unless referred by a public GP, except in emergencies. Nevertheless, some people use these services in the private sector.

The health survey respondents were interviewed about the frequency of their medical visits. Those who had any medical visit in the last 2 weeks were asked if the physician consulted at the most recent visit was a GP or a specialist. They were then asked if the physician whom they had consulted was in the public health system, was from a private health insurance company, or was a consultation for which the patient paid directly. In the first case, the GP or specialist visit was considered to be publicly financed, while in the latter two cases the visit was considered to be privately financed

Respondents were also asked if they had been hospitalised for at least one night in the year preceding the interview. Those who had been hospitalised were then asked if the expense of the last hospital admission had been covered by any type of public health insurance, by any private health insurance or had been paid directly by the patient. In the first case the hospitalisation was considered to be publicly financed, while in the later two cases it was considered to be privately financed. They were also asked about the reason for the last hospital admission: surgery, diagnostic study, medical treatment, birth and others. For purposes of the present analysis we excluded hospital admissions for birth.

All these questions about the use of health services are included in the six national health interview surveys that have been carried out in Spain since 1987. During this period the results observed have been consistent both among the various national surveys and with the results of regional health surveys that have used a modified formulation of the questions.

The variables reflecting socioeconomic position were educational level of the respondent, social class of the reference person in the household, and household economic income (table 1) [17]. The non-response rate for the question on household economic income was 26%. To reduce this percentage, an income value was imputed for non-respondents using a modification of the hot-deck imputation procedure proposed by Cox and Cohen [18]. Following this procedure, respondents who answered the question on income were classified according to a combination of the following variables: age (in 10-year intervals), sex, educational level and social class. The most frequent value for income in each of these categories was obtained, and was applied to respondents with missing income data who were in the same category after grouping them according to the same variables.

**Table 1:** Definition of the indicators of socioeconomic position used in this study

**Education^1^**	**Social Class of the person of reference^2^**	**Monthly household income^3^**
**Tertiary education**	**I–II**	**More than 1800 euros**
Second stage of tertiary education	Legislators, senior officials and mangers	More than 6000 euros
First stage of tertiary education	Professionals	3601 to 6000 euros
	Technicians and associated professionals	1801 to 3600 euros
**Upper secondary education**		
	**III**	**1201 a 1800 euros**
**Lower secondary, or second stage of basic education**	Clerks	
	Administrative personnel	**901 a 1200 euros**
**No education or primary education**	Service and sales workers	
Primary education or first stage of basic education	Self-employed	**Up to 900 euros**
Pre-primary education	Supervisors	601 to 900 euros
No education at all		361 to 600 euros
	**IV**	Less than 360 euros
	Skilled manual workers	
	**V–VI**	
	Semi-skilled manual workers	
	Unskilled manual workers	

1. Based on highest academic diploma received.

2. The question referred to current or last occupation held by the person of reference (the one who contributed the largest income to the household). Occupation was coded according to the 1994 National Classification of Occupations and was assigned to a social class based on the classification of the Spanish Society of Epidemiology^17^.

3. The question referred to the monthly income of all household members, whatever the source of the income, after tax deductions. Respondents had to choose one alternative from eight income ranges.

The variable selected to represent the need for health care was the number of chronic diseases reported by respondents during the survey. They were asked whether a physician had ever told them they suffered from any of a list of 16 chronic diseases (response categories: yes/no). The replies to these 16 questions were grouped into a single variable with the following four categories: none, 1, 2–3 and 4 or more chronic diseases.

### Statistical analysis

We first calculated the frequency – in percentage – of the use of each health service – public and private – in each category of the measures of socioeconomic position. We then estimated the magnitude of the association between the measures of socioeconomic position and the use of each health service. For this analysis, binomial regression was used to calculate the percentage ratio, taking the category that reflected the highest socioeconomic position as the reference group [19]. The variables age, sex and number of chronic diseases were included in the regression models due to their potential confounding effect and/or because they were indicators of the need for care. In Spain, as in most of the developed countries, persons in low socioeconomic position have a higher prevalence of a large variety of chronic diseases than persons in high socioeconomic position [20,21]. In the case of household economic income, the number of persons residing in the household was also included as a control variable.

Persons in low socioeconomic position showed higher frequency of majority of the chronic diseases, such as hypertension, diabetes, asthma and chronic obstructive pulmonary diseases, heart diseases, stomach ulcer, muscolo-skeletal problems or hernias. Nevertheless, since some chronic diseases (like allergies) are more frequent in persons in high socioeconomic position, we evaluated the possible interaction between each measure of socioeconomic position and the number of chronic diseases. However, the interaction term was not significant in any of these analyses.

An additional analysis was performed to determine if the data source used provided estimates similar to those of previous studies. For this purpose we studied the magnitude of the association between the measures of socioeconomic position and the use of any of these health services, without differentiating between public and private services.

## Results

Some 19.8% of respondents had visited a GP, and 6.9% had seen a specialist in the 2 weeks before the interview, while 7.9% had been admitted to hospital in the preceding year. Table 2 shows the frequency of health services use overall and by socioeconomic position. Most GP consultations and almost 90% of hospitalisations were public, whereas slightly more than one fourth of the specialist visits were made with private financing. The magnitude of the frequency in the use of the public services showed an inverse gradient with socioeconomic position in all three cases, whereas exactly the opposite gradient was seen in the use of private services.

**Table 2:** Sample size, frequency (in percentage) of GP and specialist visits in the 2 weeks before the interview and frequency (in percentage) of hospitalisation during the year before the interview: total and by three indicators of socioeconomic position

**Measures of socioeconomic position**	Sample size	**GP visits**		**Specialist visits**		**Hospitalisation**	
		
		Public	Private	Public	Private	Public	Private
	
Total	18,837	18.8	1.0	5.0	1.9	6.9	1.0
							
**Education**							
Tertiary	2,870	9.8	2.4	3.3	4.4	4.1	1.9
Upper secondary	4,335	13.3	1.1	4.4	2.4	4.6	0.9
Lower secondary	3,209	17.1	0.6	5.0	1.1	6.3	0.9
Primary/no education	8,423	26.8	0.4	6.1	1.0	9.8	0.7
							
**Social class^a^**							
I–II	3,240	11.5	2.6	4.5	3.6	4.9	2.2
III	4,804	16.5	1.2	4.7	2.6	6.1	1.3
IV	5,665	22.0	0.5	5.7	1.2	7.7	0.6
V–VI	4,846	22.6	0.1	5.1	0.7	8.3	0.4
							
**Monthly income^b^**							
More than 1800 euros	3,762	11.6	2.1	4.4	3.5	4.8	1.7
From 1201 to 1800 euros	3,756	16.7	0.9	4.8	1.9	5.9	1.2
From 901 to 1200 euros	3,756	18.6	0.6	5.4	1.2	7.1	0.6
Up to 900 euros	6,234	27.8	0.4	5.5	1.0	9.8	0.3

a. No social class could be assigned to 1.5% of those interviewed.

b. No category of monthly income could be assigned to 7% of those interviewed.

The relation between socioeconomic position and use of public services is presented in table 3 After adjusting for age, sex and number of chronic diseases, a gradient was seen in the percentage ratio for public GP visits and hospitalisation, with subjects in the lowest socioeconomic position showing the largest magnitude. The percentage ratio for those in the lowest socioeconomic position ranges between 1.61 and 1.88 for GP visits and between 1.39 and 1.57 for hospitalisation. That is, for persons in the lowest socioeconomic position, the probability of going to a public GP is 61–88% higher than for those in the highest socioeconomic position, while the probability of using public hospitals is 39–57% higher. There were no significant differences in the percentage ratio for the use of public specialists, except in the case of educational level, where higher values were seen for all three categories of less than tertiary education.

**Table 3:** Use of public health services by three indicators of socioeconomic position. Percentage ratio (RP) and 95% confidence interval (CI)

**Measures of socioeconomic position**	**GP visits**				**Specialist visits**				**Hospitalisation**			
	
	Model 1^a^		Model 2^b^		Model 1^a^		Model 2^b^		Model 1^a^		Model 2^b^	
	RP (IC 95%)		RP (IC 95%)		RP (IC 95%)		RP (IC 95%)		RP (IC 95%)		RP (IC 95%)	
	
**Education**												
Tertiary	1.00	-	1.00	-	1.00	-	1.00	-	1.00	-	1.00	-
Upper secondary	1.44	(1.27–1.63)	1.39	(1.23–1.58)	1.42	(1.13–1.78)	1.37	(1.09–1.72)	1.18	(0.96–1.45)	1.13	(0.92–1.39)
Lower secondary	1.77	(1.56–2.01)	1.67	(1.47–1.89)	1.55	(1.22–1.96)	1.48	(1.16–1.879	1.60	(1.30–1.97)	1.50	(1.22–1.84)
Primary/no education	2.10	(1.27–1.63)	1.88	(1.68–2.11)	1.51	(1.22–1.88)	1.34	(1.07–1.69)	1.79	(1.49–2.16)	1.57	(1.30–1.90)
												
**Social class**												
I–II	1.00	-	1.00	-	1.00	-	1.00	-	1.00	-	1.00	-
III	1.33	(1.19–1.48)	1.29	(1.6–1.43)	0.99	(0.82–1.21)	0.97	(0.80–1.18)	1.16	(0.99–1.38)	1.14	(0.95–1.36)
IV	1.81	(1.63–2.00)	1.68	(1.52–1.85)	1.24	(1.04–1.49)	1.18	(0.98–1.41)	1.49	(1.26–1.76)	1.41	(1.20–1.67)
V–VI	1.83	(1.66–2.03)	1.72	(1.55–1.90)	1.09	(0.90–1.32)	1.03	(0.85–1.25)	1.61	(1.36–1.91)	1.53	(1.29–1.81)
												
**Monthly income**												
More than 1800 euros	1.00	-	1.00	-	1.00	-	1.00	-	1.00	-	1.00	-
From 1201 to 1800 euros	1.41	(1.28–1.56)	1.38	(1.25–1.52)	1.06	(0.89–1.26)	1.03	(0.87–1.23)	1.20	(1.02–1.41)	1.21	(1.02–1.41)
From 901 to 1200 euros	1.47	(1.33–1.63)	1.38	(1.25–1.52)	1.12	(0.92–1.35)	1.05	(0.87–1.27)	1.27	(1.07–1.50)	1.27	(1.07–1.50)
Up to 900 euros	1.80	(1.64–1.98)	1.61	(1.47–1.77)	0.96	(0.79–1.16)	0.87	(0.87–1.05)	1.39	(1.19–1.63)	1.39	(1.19–1.63)

a. Adjusted for age and sex

b. Adjusted for age, sex, and number of chronic diseases. In monthly income, also adjusted for number of members in household.

The percentage ratio in the use of private services showed a gradient in all three cases, with subjects in the highest socioeconomic position presenting the greatest magnitude (table 4). After adjusting for age, sex and number of chronic diseases, the percentage ratio for subjects in the lowest socioeconomic position ranged between 0.03 and 0.11 for GP visits, between 0.16 and 0.24 for specialist visits, and between 0.14 and 0.25 for hospitalisation. That is, the probability of persons in the lowest socioeconomic position using a private service was 74–97% lower than for those in the highest socioeconomic position.

**Table 4:** Use of private health services by three indicators of socioeconomic position. Percentage ratio (RP) and 95% confidence interval (CI)

**Measures of socioeconomic position**	**GP**				**Specialist**				**Hospitalisation**			
	
	Model 1^a^		Model 2^b^		Model 1^a^		Model 2^b^		Model 1^a^		Model 2^b^	
	RP (IC 95%)		RP (IC 95%)		RP (IC 95%)		RP (IC 95%)		RP (IC 95%)		RP (IC 95%)	
	
**Education**												
Tertiary	1.00	-	1.00	-	1.00	-	1.00	-	1.00	-	1.00	-
Upper secondary	0.49	(0.34–0.68)	0.48	(0.34–0.67)	0.58	(0.45–0.73)	0.56	(0.44–0.71)	0.55	(0.38–0.80)	0.54	(0.37–0.79)
Lower secondary	0.25	(0.16–0.40)	0.24	(0.15–0.39)	0.26	(0.19–0.37)	0.25	(0.18–0.25)	0.49	(0.32–0.74)	0.47	(0.30–0.71)
Primary/no education	0.12	(0.08–0.18)	0.11	(0.07–0.17)	0.18	(0.14–0.24)	0.16	(0.12–0.22)	0.28	(0.19–0.42)	0.25	(0.17–0.38)
												
**Social class**												
I–II	1.00	-	1.00	-	1.00	-	1.00	-	1.00	-	1.00	-
III	0.45	(0.32–0.62)	0.44	(0.32–0.62)	0.74	(0.58–0.94)	0.73	(0.57–0.92)	0.58	(0.42–0.81)	0.56	(0.41–0.80)
IV	0.21	(0.14–0.31)	0.20	(0.14–0.31)	0.33	(0.25–0.44)	0.32	(0.23–0.41)	0.25	(0.16–0.37)	0.24	(0.16–0.36)
V–VI	0.03	(0.01–0.09)	0.03	(0.01–0.08)	0.21	(0.14–0.30)	0.20	(0.14–0.29)	0.20	(0.13–0.33)	0.19	(0.12–0.31)
												
**Monthly income**												
More than 1800 euros	1.00	-	1.00	-	1.00	-	1.00	-	1.00	-	1.00	-
From 1201 to 1800 euros	0.42	(0.30–0.59)	0.42	(0.30–0.59)	0.56	(0.44–0.71)	0.55	(0.44–0.70)	0.64	(0.47–0.88)	0.63	(0.46–0.87)
From 901 to 1200 euros	0.25	(0.16–0.39)	0.24	(0.15–0.38)	0.35	(0.26–0.48)	0.34	(0.25–0.47)	0.30	(0.19–0.48)	0.29	(0.18–0.46)
Up to 900 euros	0.11	(0.06–0.18)	0.10	(0.06–0.18)	0.26	(0.19–0.36)	0.24	(0.17–0.34)	0.16	(0.09–0.27)	0.14	(0.09–0.25)

a. Adjusted for age and sex

b. Adjusted for age, sex, and number of chronic diseases. In monthly income, also adjusted for number of members in household.

When public and private health services use was combined, persons in the lowest socioeconomic position had the largest percentage ratio for any GP visit and the smallest percentage ratio for any specialist visit (table 5). In contrast, no significant socioeconomic differences were observed in the percentage ratio for any hospitalisation.

**Table 5:** Use of any health service (public or private) by three indicators of socioeconomic position. Percentage ratio (PR) and 95% confidence interval (CI)

**Measures of socioeconomic position**	**GP visits**		**Specialist visits**		**Hospitalisation**	
	PR^a ^(IC 95%)		PR^a ^(IC 95%)		PR^a ^(IC 95%)	
**Education**						
Tertiary	1.00	-	1.00	-	1.00	-
Upper secondary	1.21	(1.08–1.35)	0.90	(0.77–1.06)	0.95	(0.79–1.13)
Lower secondary	1.38	(1.23–1.54)	0.77	(0.64–0.91)	1.18	(0.98–1.41)
Primary/no education	1.52	(1.37–1.68)	0.66	(0.57–0.77)	1.16	(0.99–1.37)
						
**Social class**						
I–II	1.00	-	1.00	-	1.00	-
III	1.14	(1.03–1.25)	0.85	(0.73–0.99)	0.96	(0.82–1.12)
IV	1.42	(1.30–1.55)	0.79	(0.69–0.91)	1.06	(0.92–1.22)
V–VI	1.43	(1.30–1.56)	0.67	(0.57–0.78)	1.12	(0.97–1.30)
						
**Monthly income**						
More than 1800 euros	1.00	-	1.00	-	1.00	-
From 1201 to 1800 euros	1.24	(1.13–1.35)	0.81	(0.50–0.68)	1.05	(0.91–1.21)
From 901 to 1200 euros	1.21	(1.11–1.33)	0.72	(0.63–0.85)	1.01	(0.87–1.18)
Up to 900 euros	1.39	(1.27–1.51)	0.58	(0.71–0.93)	1.06	(0.92–1.22)

a. Adjusted for age, sex, and number of chronic diseases. In monthly income, also adjusted for number of members in household.

## Discussion

### Main findings

The use of public GP and hospital services tends to favour the worst off, which suggests pro-poor horizontal equity in both types of health services while, in general, no socioeconomic differences were found in the use of public specialist care, which suggests horizontal equity in this health service. Persons in high socioeconomic position make more frequent use of private services.

### Study limitations

Our findings with regard to health services use when no distinction was made between private and public services are similar to the results of other investigations in the general population in Spain, which supports the validity of the data source used [5,7,22]. The similarity of the findings for all three indicators of socioeconomic position supports the validity of the variable economic income, which was imputed for one fifth of respondents as a function of other variables.

The evaluation of equity in health services use requires knowledge of both the supply and quality of public health services, however, this study offers no information about these characteristics. It might be suggested that disadvantaged areas have a more limited supply of public health services, as some authors have noted [23]. However, a previous study found no differences in the use of public health services by per capita income of the area of residence [24]. Furthermore, our results in persons of low socioeconomic position, who are concentrated in these areas, suggest that this is not so.

One indicator of the quality of public health services is the level of use of private services. The results shown in table 1 suggest that most of the population supports the public system: although 28% of the specialist consults are private, only 5% of the GP consults and 13% of hospitalisations were made with private financing. These results reflect the opinion of the Spanish population about the quality of the public and private health systems. According to the most recent health opinion survey, 70% of Spaniards would choose the private system based on considerations of how rapidly patients receive care, and 80% would choose the public system if the decision is based on the availability of medical technology and the quality of physician training [25].

### Possible explanations

About 14% of the Spanish population has complementary private health insurance; this proportion ranges from 5–8% for persons in the lowest socioeconomic position to 27–28% for those in the highest, depending on the indicator of socioeconomic position used [16]. These data, together with the different economic ability to pay directly for medical services, may explain why the use of private medical care is more frequent in persons of high socioeconomic position. The similarity of the socioeconomic gradient for the three health services studied supports the idea that complementary private insurance and/or direct payments would not themselves be responsible for the socioeconomic pattern observed when private and public specialist visits are studied together. This pattern probably reflects the larger proportion represented by the use of private services in the case of specialist care.

Our results regarding public GP visits and hospital admissions could be attributed to residual confounding due to the lack of adequate measures of the need for care. However, little change was seen when we added a variable that reflects limitation of activity due to health problems (data not shown). Van Doorslaer et al also found that inclusion of a much larger battery of health measures had little effect on the relation between income and health services use [5,7].

Factors other than the existence of a health problem may be influencing the demand for public GP services, such as patients' search for solutions to social and economic problems or their need for information about bureaucratic procedures to access the public health system [26]. That is, part of the use of the public GP by persons in low socioeconomic position may be unnecessary from the health point of view.

In contrast, the findings about public hospitalisation are surprising, since the decision to hospitalise a patient depends more on the physician's recommendation than on the patient's demand. This socioeconomic gradient in public hospital admissions may be due to the opposite socioeconomic gradient observed in private hospital admissions. Persons in high socioeconomic position probably have a lower frequency of public admission because a large proportion choose private admissions.

GPs in the public sector resolve many of their patients' health problems, and this may have contributed to the lack of socioeconomic differences in the frequency of visits to public sector specialists according to social class or income. Nevertheless, this lack of socioeconomic differences is probably due to the large proportion of persons who use private specialist care, not only among persons in high socioeconomic position, but in all socioeconomic groups. In the case of persons with higher educational level, the frequency of use of private specialists is very high: almost three-fifths of consultations. The large proportion of persons in this group who choose private specialist care would explain why the rest of the categories of educational level have a significantly higher frequency of visits to specialist physicians in the public system.

When all visits to the specialist physician are taken into account (table 5), the percentage ratio in persons in the lowest socioeconomic position versus those in the highest socioeconomic position ranges between 0.58 and 0.67. This lower use of specialist physician services by persons in low socioeconomic position may be related with insufficient resources to meet the demand in the public health system, or with long waiting times for specialist physician visits in the public system, or with other reasons unrelated to the supply of services and performance of the public health system. This finding raises important ethical and policy dilemmas. Indeed, based on these results, can we consider that the higher frequency of specialist physician visits among persons with higher income means there is social inequality in specialist visits in Spain? Or must we take into account the amount of resources and performance of the public health system before making a moral judgment in this matter? And if it turns out that the public system has sufficient resources and performs adequately, should the higher frequency of specialist physician visits in subjects with higher income be a source of concern in health policy?

Almost 30% of specialist medical visits are made to private specialists. This large proportion of visits to the private specialist physician could be related with a lack of specialists in the public health system, however the results obtained do not support this as a plausible explanation. Almost all GP visits and most hospitalisations take place in the Spanish public health system, therefore it is highly unlikely to be unbalanced, with a scarcity of specialist physicians versus an adequate supply of GPs and hospital physicians. Furthermore, Spain is one of the EU countries with the highest ratio of number of public specialist visits versus public GP visits [12]. The high percentage of private visits is probably related with the waiting time for appointment with the public specialist physician, which many patients are not willing to tolerate, as reflected in the 2006 health opinion survey [25]. Thus, patients seek direct access to the specialist through complementary insurance plans or by direct payment [27].

UK citizens have also been found to rate the quality of the public healthcare system as low due to long waiting lists [28]. But this evaluation does not mean that it is rational from the health point of view and, even less, from the standpoint of assignment of public resources. For some authors, many of the deficiencies attributed to the public system are related with other factors, such as refusal of the public and the mass media to accept that the health system can resolve or improve many health problems but is less effective in dealing with others, or the naivety of thinking that the interests of medical professionals will necessarily always coincide with those of the public [29].

In any event, no UK population group can be identified that uses only private medical services for which there is an alternative in the NHS [30]. The situation is similar in Spain, given the small proportion of the population that uses private GPs and hospitals. The low use of private GPs may be due to their relative lack in private practice in general medicine and to the fact that health insurance companies allow direct access to specialists. With regard to private hospitalisation, its low use may be due to the fact that half of public hospital admissions are made via the emergency service [16], which allows patients to avoid the waiting time of programmed admissions. Furthermore, private hospitalisation is used for less serious elective admissions because high-technology care is concentred in the public sector [26].

## Conclusion

In summary, based on the definition of horizontal equity as "equal use for equal need", this study demonstrates the existence of inequity in GP visits and hospitalisation, favouring the lower socioeconomic groups, and equity in the use of the specialist physician. Future studies should aim to determine to what extent this inequity represents an overuse of public healthcare services or is due to persons of high socioeconomic position choosing to use private healthcare services.

## Competing interests

The authors declare that they have no competing interests.

## Authors' contributions

ER originated the study. DM and MEC contributed to the analysis of this study. PA, PO, and VD contributed to the drafting of the paper. ER coordinated the writing of the article. All authors contributed to the final version of the article. All authors read and approved the final manuscript. ER is the the guarantor of the article.

## Pre-publication history

The pre-publication history for this paper can be accessed here:


